# Kinetics and mechanism of jack bean urease inhibition by Hg^2+^

**DOI:** 10.1186/1752-153X-6-154

**Published:** 2012-12-10

**Authors:** Nana Du, Mingming Chen, Zhaodi Liu, Liangquan Sheng, Huajie Xu, Shuisheng Chen

**Affiliations:** 1 Fuyang Normal College, College of Chemistry and Chemical Engineering, Fuyang, 236037, People’s Republic of China; 2 Anhui University, College of Chemistry and Chemical Engineering, Hefei, 230039, People’s Republic of China

**Keywords:** Jack bean urease, Hg^2+^ ion, Inhibition, Kinetics

## Abstract

**Background:**

Jack bean urease (EC 3.5.1.5) is a metalloenzyme, which catalyzes the hydrolysis of urea to produce ammonia and carbon dioxide. The heavy metal ions are common inhibitors to control the rate of the enzymatic urea hydrolysis, which take the Hg^2+^ as the representative. Hg^2+^ affects the enzyme activity causing loss of the biological function of the enzyme, which threatens the survival of many microorganism and plants. However, inhibitory kinetics of urease by the low concentration Hg^2+^ has not been explored fully. In this study, the inhibitory effect of the low concentration Hg^2+^ on jack bean urease was investigated in order to elucidate the mechanism of Hg^2+^ inhibition.

**Results:**

According to the kinetic parameters for the enzyme obtained from Lineweaver–Burk plot, it is shown that the *K*_m_ is equal to 4.6±0.3 mM and *V*_m_ is equal to 29.8±1.7 μmol NH_3_/min mg. The results show that the inhibition of jack bean urease by Hg^2+^ at low concentration is a reversible reaction. Equilibrium constants have been determined for Hg^2+^ binding with the enzyme or the enzyme-substrate complexes (*K*_i_ =0.012 μM). The results show that the Hg^2+^ is a noncompetitive inhibitor. In addition, the kinetics of enzyme inhibition by the low concentration Hg^2+^ has been studied using the kinetic method of the substrate reaction. The results suggest that the enzyme first reversibly and quickly binds Hg^2+^ and then undergoes a slow reversible course to inactivation. Furthermore, the rate constant of the forward reactions (*k*_+0_) is much larger than the rate constant of the reverse reactions (*k*_-0_). By combining with the fact that the enzyme activity is almost completely lost at high concentration, the enzyme is completely inactivated when the Hg^2+^ concentration is high enough.

**Conclusions:**

These results suggest that Hg^2+^ has great impacts on the urease activity and the established inhibition kinetics model is suitable.

## Background

Urease (urea amidohydrolase, EC 3.5.1.5) is a nickel-containing enzyme that catalyzes the hydrolysis of urea to ammonia and carbon dioxide. The enzyme is widely distributed in nature and is found in a variety of plants, algae, fungi, bacteria and soil [1-3]. Thus, urease plays an important role in the nitrogen metabolism of many microorganism and plants [4,5]. Despite the great importance of urease in biotechnology and medicine and also continuous interest in many laboratories [6-11], the mechanisms of hydrolysis of the metal active site of the enzyme have not been studied in detail [12]. Study show that the cysteine is involved in the catalysis, as was demonstrated by reacting the enzyme with cysteine-reactive agents [13,14]. In view of the complexity of the role of the active site flap cysteine in the urease catalysis, effect of potential inhibitors on the reactivity of enzyme thiol groups, the active site flap thiol in particular, was investigated [15,16].

The main classes of urease inhibitors are: boroncontaining compounds [7], thiol compounds [13], phosphate [15], phosphoroamide compounds [17], hydroxamic acids [14], F^−^ ions [18], and heavy metal ions [19,20]. Among the known inhibitors of urease, the inhibition of urease by heavy metal ions is said to result from the reaction of these ions with a sulfhydryl group in the active center of the enzyme by a reaction analogous to the formation of metal sulfides [21]. The formation of sulfides with the active center was confirmed experimentally by the correlation between the inhibitory efficiency of metal ions and the solubility products of their sulfides [22]. So, inhibition of urease by heavy metal ions can be studied to investigate the effect on the reactivity of enzyme thiol groups. Moreover, the inhibition of heavy metal ions is related to its biological toxicity, which causing loss of the biological function of the enzyme then affects the growth and survival of the animal and plants [20,22]. Studies published so far on inhibition of urease of both plant and bacterial origin by heavy metal ions have aimed either at investigating their toxicity or at detection of their trace amounts, e.g. of Hg^2+^, Cu^2+^ and Ag^+^ ions [23-27]. Mercury is a known biotoxicant that can accumulate in the human body and show up in the food chain [28]. However, inhibitory kinetics of urease by low concentration Hg^2+^ has not been explored fully and only a few studies on the urease inhibition activities of heavy metal ions have appeared in the literature.

In this study, the inhibitory effect of the low concentration Hg^2+^ on jack bean urease was investigated. Our present investigation not only studied the kinetics of inhibition of the jack bean urease by the low concentration Hg^2+^ specifically but also researched related inhibition mechanisms. The results show that Hg^2+^ has great impacts on the jack bean urease activity. The inhibition of jack bean urease by the low concentration Hg^2+^ is shown to be reversible. In addition, the rate constants of inactivation are determined.

## Results

### Effect of Hg^2+^ concentration on urease activity

**Figure 1:**
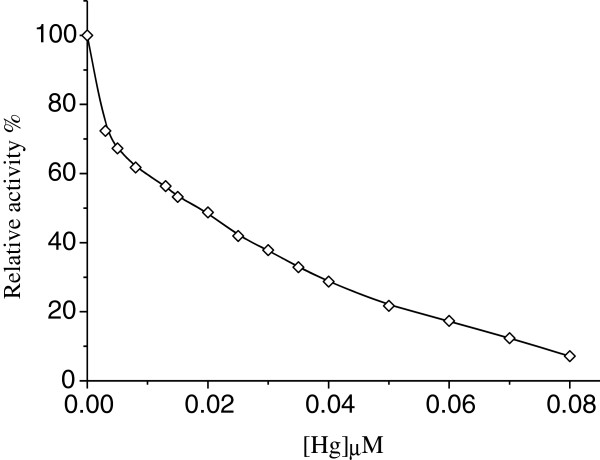
**Effect of Hg**^**2+**^**on jack bean urease activity.** Conditions were 1 mL assay system containing 0.02 M KH_2_PO_4_- K_2_HPO_4_ buffer (pH 7.4), 0.67 mM of urea, 6 μg ml^-1^ of enzyme and different concentrations of Hg^2+^ at 25°C for 15 min.

Urease (6 μg/ml, 0.02 M KH_2_PO_4_-K_2_HPO_4_) was incubated with different concentrations of Hg^2+^ for 15 min. Then, mixed solutions of urease and Hg^2+^ were added into the assay media (0.67 mM urea) to investigate the relation of Hg^2+^ concentration and the residual activity (Figure 1). In certain range, the activity of the enzyme decreases with increase of Hg^2+^ concentration. The *IC*_50_ value is estimated to be 0.018±0.001 μM when the Hg^2+^ concentration could lead to 50% of enzyme activity loss. The enzyme activity is almost completely lost when the Hg^2+^ concentration reach 0.08 μM.

### The inhibition mechanism of urease by Hg^2+^

**Figure 2:**
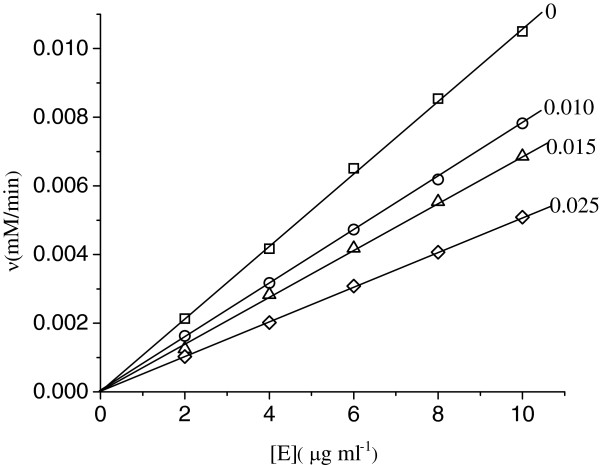
**The relationship between the enzyme activity and urease concentration.** The assay conditions were as described for Figure 1, except that the enzyme concentration. Numbers denote Hg^2+^ concentration (μM).

The inhibition mechanism on the urease by Hg^2+^ was studied. The procedure used to obtain relationship between the enzyme activity and urease concentration was same as in the residual activity experiment. Figure 2 shows the relationship between the enzyme activity and urease concentration. The plots of the remaining enzyme activity against the enzyme concentrations in the presence of different Hg^2+^ concentrations give a family of straight lines, which all pass through the origin, indicating that the inhibition of Hg^2+^ on the enzyme is reversible [29]. Increasing the inhibitor concentration resulted in a descending slope of the line. The presence of inhibitor does not bring down the amount of effective enzyme, but just result in the inhibition and decreasing of activity of the enzyme.

**Figure 3:**
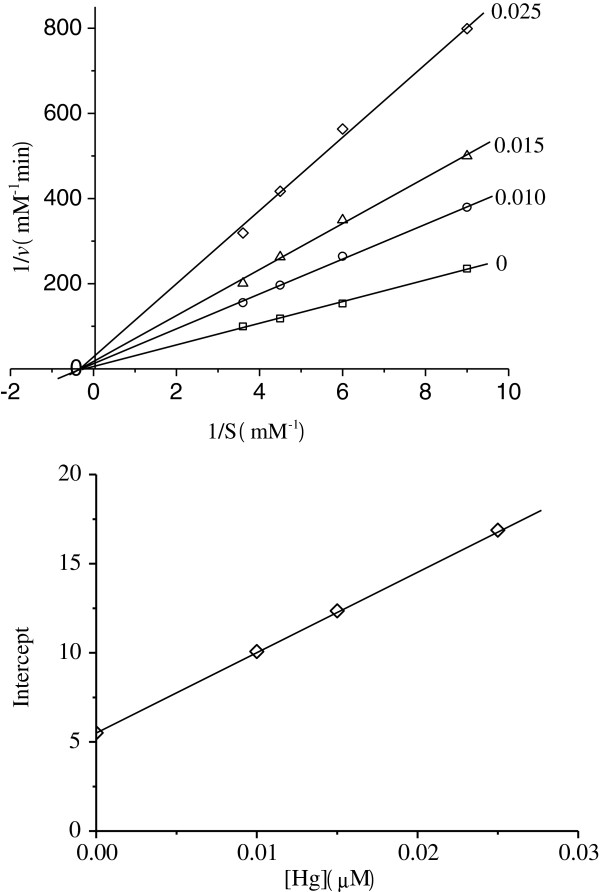
**Plots of 1/*v *vs. 1/[S].** The assay conditions were as described for Figure 1. Numbers denote Hg^2+^ concentration (μM). The insets represent the plot of the intercept (1/*V*_m_) vs. the Hg^2+^ concentrations to determine the inhibition constant, *K*_i_.

In this section, the Hg^2+^ concentration was held at different constant values (lower than for complete inactivation), while the substrate concentrations were varied, permitting measurement of the effect of increasing substrate concentration [S] on the initial reaction rate (*v*). Plots of 1/*v* vs. 1/[S] for different concentrations of inhibitor (Figure 3) show that Hg^2+^ is a noncompetitive inhibitor [30]. Increasing the Hg^2+^ concentration results in a family of lines with a common intercept on the 1/[S] axis but with different slopes and intercepts on the 1/*v* axis, indicating that *K*_m_ is unchanged by the presence of different inhibitor concentrations while *V*_m_ is changed. The equilibrium constant for the inhibitor binding, *K*_i_, can be obtained from the plot of 1/*V*_m_ versus the Hg^2+^ concentration, as shown in the inset. The inhibition constant, *K*_i_, for Hg^2+^ obtained from the experimental data is 0.012 μM.

### Determination of the kinetic parameters of urease

The kinetic behavior of jack bean urease in the hydrolysis of urea has been studied specifically. Under the conditions employed in the present study, the hydrolysis of urea by urease follows Michaelis-Menten kinetics. The kinetic parameters for the enzyme have been obtained from Lineweaver–Burk plot (Figure 3, curve 0) with *K*_m_ (4.6±0.3 mM) and *V*_m_ (29.8±1.7 μmol NH_3_/min mg) which are in agreement with previously obtained values [31,32].

### Kinetics of the substrate reaction in the presence of different concentrations of Hg^2+^

**Figure 4:**
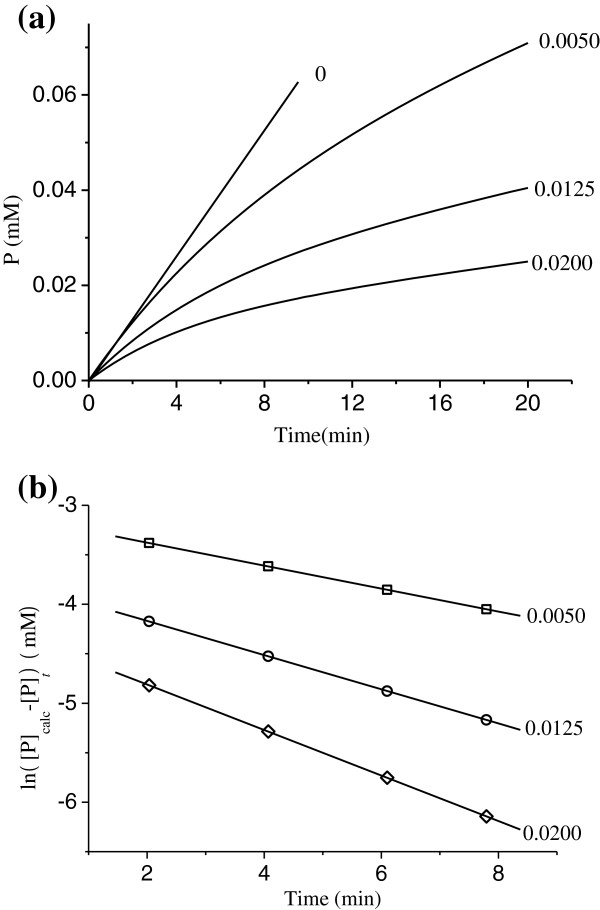
**Inhibition kinetics for jack bean urease in various concentrations of Hg**^**2+**^**.** (**a**) Course of substrate reaction. Assay conditions were as described for Figure 1. Numbers denote Hg^2+^ concentration (μM). (**b**) Semilogarithmic plot of ln([P]_calc_-[P]_*t*_) vs. *t*, the data was taken from curves in (**a**).

Figure 4(a) shows the time process of the hydrolysis of the substrate without enzyme–inhibitor preincubation. The assay mixture (1.0 ml) contained 40 mg L^-1^ urea, 6 μg ml^-1^ urease and different concentrations Hg^2+^ in 0.02 M KH_2_PO_4_-K_2_HPO_4_ buffer (pH=7.4). At each Hg^2+^ concentration, the rate decreases with increasing time until a straight line is approached, is accord with the conclusion in ref.22. The results analyzed by Tsou′s method [33] suggest that the formation of the enzyme Hg^2+^ complex is a reversible reaction at low Hg^2+^ concentration [29], while the formation of the enzyme Hg^2+^ complex is irreversible reaction at 1.0 μM Hg^2+^ because the asymptote becomes to parallel to abscissa without residual enzyme activity [22]. In this paper, our chief aim is to investigate the reversible inhibition kinetics of the enzyme by Hg^2+^ at low concentration. According to Eq. (6), plots of ln([P]_calc_-[P]_*t*_) versus *t* give a family of straight lines at different concentrations of Hg^2+^ with slopes of –*A* (Figure 4(b)), the apparent rate constants of inhibition. The microscopic rate constant of the reverse inactivation of the enzyme (*k*_-0_) in these experimental conditions are listed in Table 1. The value of *k*_-0_ is almost the same for different Hg^2+^ concentrations. From Eq. (7), a plot of 1/(*Ak*_-0_) versus 1/[Hg] gave a straight line, which is fit using a least squares analysis. The straight line with a slope of *K*_i_/*k*_+0_ intercepts on the ordinate with 1/*k*_+0_. The microscopic rate constant, *k*_+0_, and the equilibrium constant for inhibitor binding, *K*_i_, obtained from these values is 0.013 μM.

**Table 1:** Microscopic inhibitory rate constants of the jack bean urease in different concentrations [S]

**[S] (mg L**^**-1**^**)**	***k***_**+0**_**(min**^**-1**^**)**	***k***_**-0**_**(min**^**-1**^**)**
20		0.036
30		0.037
40	0.267^I^ 0.303^II^	0.036
50		0.035

^I^ From the intercept of the straight line of 1/(*A*-*k*_-0_) vs. 1/[Hg].^II^ From the slope of the straight line of 1/(*A*-*k*_-0_) vs. 1/[Hg].

### Kinetics of the reaction at different substrate concentrations in the presence of Hg^2+^

**Figure 5:**
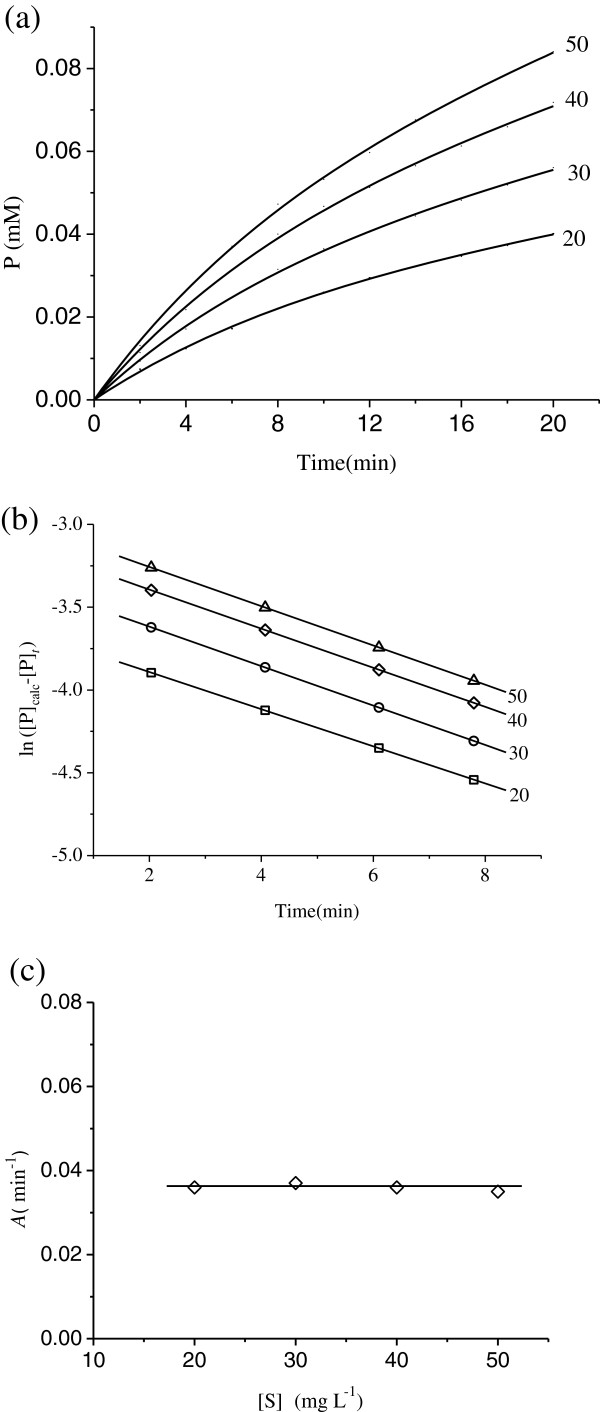
**Inhibition kinetics for jack bean urease in various concentrations of substrate.** (**a**) Course of the substrate reaction. Assay conditions were as described for Figure 1. Numbers denote urea concentration (mg L^-1^). (**b**) Semilogarithmic plot of ln ([P]_calc_ – [P]_*t*_) vs. *t* with the data taken from curves in (**a**). (**c**) The apparent forward inactivation rate constants (*A*) vs. urea concentrations in the presence of Hg^2+^.

Figure 5(a) shows the kinetic courses of substrate reaction at different urea concentrations in the presence of 0.005 μM Hg^2+^. At each substrate concentration, the rate decreases with increasing time until a straight line is approached. The initial rate and the slope of the asymptote increase with increasing substrate concentration. Similarly, plots of ln([P]_calc_-[P]_*t*_) against *t* give a family of straight lines at different concentrations of the substrate with slopes of - *A* (Figure 5(b)). It can be obtained through suitable plots for the apparent forward rate constants, *A*. A plot of the slopes of the straight lines in Figure 5(b) versus substrate concentration [S] gives a horizontal straight line, Figure 5(c), indicating that the substrate concentration [S] does not affect the microscopic rate constants: *k*_+0_ and *k*_-0_. The results are shown in Table 2.

**Table 2:** **Microscopic rate constants of jack bean urease by different concentrations Hg**^**2+**^

**[Hg] (μM)**	***A*** **(min**^**-1**^**)**	***k***_**+0**_**(min**^**-1**^**)**	***k***_**-0**_**( min**^**-1**^**)**
0.0050	0.118	0.279	0.036
0.0125	0.173	0.267	0.037
0.0200	0.228	0.307	0.036

## Discussion

In this paper, the effects of low concentration Hg^2+^ on the enzyme activity were studied specifically, the results of which show that the inhibitor concentration leading to 50% of enzyme activity loss, *IC*_50_, is estimated to be 0.018±0.001 μM. In addition, kinetics of inhibition of the enzyme by Hg^2+^ at the low concentration was also studied by the substrate reaction kinetic method described by Tsou [33]. As a result, the kinetic parameters for the enzyme obtained from Lineweaver–Burk plot show that *K*_m_ is equal to 4.6±0.3 mM and *V*_m_ is equal to 29.8±1.7 μmol NH_3_/min mg, which generally accord with the values reported in literature [31,32]. When the reaction time is sufficiently long, the concentration of product approaches a constant final value, which decreases as the concentration of Hg^2+^ increases and is accord with the conclusion in ref.22. The treated enzyme still has partial residual activity at low concentration of Hg^2+^, which is analyzed by the method described by Tsou [33] and also suggests that inactivation of urease by the low concentration Hg^2+^ is a reversible reaction under the experimental conditions [29], while the formation of the enzyme Hg^2+^ complex is an irreversible reaction at 1.0 μM Hg^2+^ because the asymptote tends to parallel to the abscissa without residual enzyme activity [22]. The plots of the remaining enzyme activity against the enzyme concentrations in the presence of Hg^2+^ with different concentrations give a family of straight lines, all of which pass through the origin, indicating that the inhibition of Hg^2+^ on the enzyme is reversible [29]. The results suggest that the enzyme first reversibly and quickly binds Hg^2+^ and then undergoes a slow reversible course to inactivation on the basis of the previous study [22]. Plots of 1/*v* against 1/[S] at different concentrations of Hg^2+^ give a family straight line with a common intercept on the 1/[S] axis but with different slopes and intercepts on the 1/*v* axis, which indicates that Hg^2+^ is a noncompetitive inhibitor for the enzyme [30].

In accordance with the microscopic rate constants are determined and shown in Table 1 and Table 2, we can see that the values of *k*_+0_ are almost the same (0.279, 0.267, 0.307 min^-1^) and the values of *k*_-0_ are the same (0.036, 0.037, 0.036 min^-1^) as well. Furthermore, the microscopic rate constants of the forward and the reverse reactions slightly vary with the increasing concentration of the substrate. The results indicate that the presence of substrate does not provide protection for the enzyme against the inhibition by Hg^2+^. Besides, the rate constant for forward inactivation (*k*_+0_) is much larger than that for reverse reactivation (*k*_-0_). According to combination with the fact that the enzyme activity is almost completely lost when the concentration of Hg^2+^ reaches 0.08 μM, the enzyme is completely inactivated when the concentration of Hg^2+^ is high enough. The results suggest that Hg^2+^ has great impacts on the urease activity. Based on the fact that thiols are involved in binding Hg that was provided in the previous study, there is another demonstration that sulfhydryl group is essential for the activity of the enzyme.

## Experimental

### Materials

Jack bean urease, Fluka, with specific activity 57 μmol NH_3_/min mg protein was used (without further purified). Urea (substrate), mercuric chloride (inhibitor) and other reagents are local products of analytical grade. The water used was redistilled and ion-free.

### Assay

The standard residual activity assay mixture (1.0 ml) contained 0.67 mM urea in 0.02 M KH_2_PO_4_-K_2_HPO_4_ buffer (pH 7.4). Urease was incubated by Hg^2+^ for 15 min. Reactions were initiated by the addition of small aliquots of the enzyme and Hg^2+^ mixed solution, typically 3 μl containing 2 mg ml^-1^ enzyme. After reaction for 15 at 25°C, 2 ml phenol solution and 3 ml hypochlorite sodium solution were added into the mixture at 35°C.

Progress curves of the enzyme reactions were obtained by measuring the ammonia generated as a function of reaction time. The assay mixture (1.0 ml) typically contained 20-50 mg L^-1^ urea in 0.02 M KH_2_PO_4_-K_2_HPO_4_ buffer (pH 7.4), 0.005–0.025 μM Hg^2+^ and 6 μg ml^-1^ urease. Reactions were initiated by adding urease to the mixture of urea and Hg^2+^. After reaction for some time at 25°C, 2 ml phenol solution and 3 ml hypochlorite sodium solution were added into the mixture at 35°C.

The reaction was monitored by measuring the ammonia concentration by the phenol-hypochlorite method [34] in samples, which were removed from the reaction mixtures at time intervals. Absorption at 625 nm was recorded using a spectrophotometer. One unit (U) of enzymatic activity was defined as the amount of enzyme required to produce 1μM ammonia per min under these conditions. The activity of uninhibited urease was accounted as the control activity of 100%.

### Determination of microscopic rate constants

The progress-of-substrate-reaction method that previously described by Tsou [33] was used to study the inhibitory kinetics of jack bean urease by Hg^2+^. The substrate reaction progress curve was analyzed to obtain the reaction rate constants as detailed below. The reaction was carried out at a constant temperature of 25°C.

**Scheme 1:**
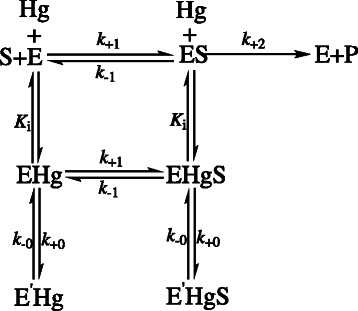
The kinetic model of the enzyme reacting with the substrate and the inhibitor.

For slow, reversible inhibition with fractional residue activity, the kinetic model of the enzyme reacting with the substrate and the inhibitor can be written as Scheme 1[30], where S, P, Hg and E respectively denote substrate, product, inhibitor (Hg^2+^) and enzyme. EHg, ES and ESHg are the respective complexes. E'Hg and E'HgS are inactive enzyme forms. *K*_i_ is the inhibition constant for the inhibitor (Hg^2+^). *k*_+0_ and *k*_-0_ are rate constants for forward and reverse inactivation of the enzyme, respectively.

As is usual the case [S]» [E_0_] and that the modification reactions are relatively slow compared with the set up of the steady-state of the enzymatic reaction. The product formation can be written as:

(1) $${\left[P\right]}_{t}=\frac{vk_{-0}}{A}t+\frac{\left(A-k_{-0}\right)v}{A^{2}}\left(1-e^{-\mathrm{At}}\right)$$

(2) $$A=\frac{k_{+0}\left[\mathrm{Hg}\right]}{K_{i}+\left[\mathrm{Hg}\right]}+k_{-0}$$

(3) $$B=k_{-0}$$

where [P]_*t*_ is the concentration of the product formed at time *t*, which is the reaction time. *A* and *B* are the apparent rate constants for inactivation and reactivation, respectively. *k*_+0_ and *k*_-0_ are the microscopic rate constants for the forward and reverse reactions, [S] and [Hg] are the concentrations of the substrate and inhibitor. *v* is the initial rate of reaction in the absence of the inhibitor, where *v*=*V*_m_×[S]/(*K*_m_+[S]), which is the Michaelis-Menten equation. When *t* is sufficiently long, the curves become straight lines and the product concentration is written as [P]_calc_:

(4) $${\left[P\right]}_{\mathrm{calc}}=\frac{vk_{-0}}{A}t+\frac{\left(A-k_{-0}\right)v}{A^{2}}$$

A plot of [P]calc vs. *t* gives a straight line. Combining Eqs. (1) and (4) gives:

(5) $${\left[P\right]}_{\mathrm{calc}}-{\left[P\right]}_{t}=\frac{\left(A-k_{-0}\right)v}{A^{2}}e^{-\mathrm{At}}$$

(6) $$\ln\left({\left[P\right]}_{\mathrm{calc}}-{\left[P\right]}_{t}\right)=\ln\left[v\left(A-k_{-0}\right)/A^{2}\right]-\mathrm{At}$$

where [P]_calc_ is the product concentration to be expected from the straight-line portions of the curves as calculated from Eq. (4) and [P]_*t*_ is the product concentration actually observed at time *t*. Plots of ln([P]_calc_-[P]_*t*_) versus *t* give a series of straight lines at different concentrations of inhibitor ([Hg]) with slopes of –*A*. The apparent forward rate constant *A* can be obtained from such graphs. From Eq. (4), a plot of [P]_calc_ against time, *t*, gives a straight line with a slope of *vk*_-0_/*A*. From the slope of the straight line, *k*_-0_ can be obtained.

The apparent forward rate constant, *A*, is independent of the substrate concentration, but depends on the inhibitor concentration From Eq. (2) and Eq. (3):

(7) $$\frac{1}{A-k_{-0}}=\frac{K_{i}}{k_{+0}\left[\mathrm{Hg}\right]}+\frac{1}{k_{+0}}$$

Plot of 1/(*A*-*k*_-0_) versus 1/[Hg] gives a straight line with a slope of *K*_i_/ *k*_+0_ and an intercept of 1/*k*_+0_ on the ordinate and -1/*K*_i_ on the abscissa which can be used to determine the microscopicrate constant, *k*_+0_, and the equilibrium constant for the inhibitor binding, *K*_i_.

## Conclusions

The inhibitory effect of Hg^2+^ on jack bean urease was investigated that the Hg^2+^ displayed strong inhibitory activity against jack bean urease. The experiment result shows that the inhibition of urease by low concentration Hg^2+^ is a reversible reaction and the inhibition belongs to be noncompetitive. The microscopic rate constants of the forward and the reverse reactions vary little with increasing substrate concentration, indicating that presence of substrate does not offer protection of this enzyme against inhibition by Hg^2+^. The data fit well to model, which indicates that the established inhibition kinetics model is suitable.

## Competing interests

The authors declare that they have no competing interests.

## Authors’ contributions

ND made a significant contribution to acquisition of data, analysis and manuscript preparation. MC has made a substantial contribution to experimental design and data analysis. ZL and HX participated in partial experiments. LS made a significant contribution to experimental design, data analysis, and manuscript revision. SC participated in study design and manuscript revision. All authors read and approved the final manuscript.
